# Supplementary material for: Postharvest Treatment with Abscisic Acid Alleviates Chilling Injury in Zucchini Fruit by Regulating Phenolic Metabolism and Non-Enzymatic Antioxidant System
Source: Antioxidants (Basel). 2023 Jan 16;12(1):211. doi: 10.3390/antiox12010211 (PMC9854589; doi:10.3390/antiox12010211)
Supplement: Supplementary file 1 [file antioxidants-12-00211-s001.zip › antioxidants-2143743-supplementary.pdf]

antioxidants

MDPI

Supplementary Materials

Postharvest Treatment with Abscisic Acid Alleviates Chilling Injury in Zucchini Fruit by Regulating Phenolic Metabolism and Non-Enzymatic Antioxidant System

Table S1. UPLC-MS/MS conditions for the determination of soluble phenolic compounds

Compound	Polarity	Transition 1 Quantification (*m/z*)	Cone voltage (V)	Collision energy (ev)	Transition 2 Confirmation (m/z)	Cone voltage (V)	Collision energy (ev)
**Vanillic acid**	Negative	151.14→135.59	20	10			
**Ellagic acid**	Negative	301.10→144.59	12	36	301.10→228.86	12	26
**Ferulic acid**	Negative	192.96→133.51	2	16	192.96 →177.64	2	12
**Coumaric acid**	Negative	162.82→92.94	18	22	162.82→118.95	18	10
**Quercetin**	Negative	301.07→150.63	44	20	301.07→178.67	44	14
**Isorhamnetin**	Negative	314.90→255.05	58	20	314.90→283.35	58	14
**Myricetin**	Negative	316.75→162.89	20	10	316.75→190.92	20	10
**Naringenin**	Negative	271.10→118.52	6	26	271.10→150.63	6	18
**Luteolin**	Negative	285.08→132.52	30	32	285.08→150.63	30	24
**Rhoifolin**	Negative	577.20→210.92	72	78	577.20→268.92	72	36
**Chrysoeriol**	Negative	299.10→255.92	26	28	299.10→283.94	26	20
